# Impact of same day emergency care services on urgent and emergency care delivery outcomes: a systematic review

**DOI:** 10.1136/emermed-2024-214821

**Published:** 2025-07-07

**Authors:** Katherine Jones, Christopher Carroll, Steve Goodacre, Carl Marincowitz, Anthea Sutton, Anastasios Bastounis, Andrew Booth

**Affiliations:** 1 School of Medicine and Population Health, Sheffield Centre for Health and Related Research (SCHARR), The University of Sheffield, Sheffield, UK

**Keywords:** urgent care, emergency department, Systematic Review

## Abstract

**Introduction:**

Same day emergency care (SDEC) describes an ambulatory service designed to provide an alternative to ED management, reduce admission rates and improve emergency care system performance. This systematic review aimed to identify and synthesise the evidence base for SDEC and its impact on urgent and emergency healthcare delivery.

**Methods:**

Eight bibliographic databases were searched, including: MEDLINE, EMBASE, PsycInfo, CINAHL, the Science and Social Science Citation Indices in the Web of Science Core Collection, Health Management Information Consortium, the Cochrane Library and Epistemonikos. Study selection, extraction and quality assessment were conducted independently by two reviewers. Given the clinical heterogeneity and weakness of the evidence base to determine intervention effect, a narrative synthesis was performed. Formal assessment of implementation was undertaken using the Quality Improvement Minimum Quality Criteria Set.

**Results:**

We identified 1283 citations, with 21 publications reporting 20 evaluations (18 UK and 2 Australia). SDEC services were heterogeneous in terms of referral sources, patient selection and specialties provided. Studies were mostly single centre and compared SDEC care with alternative services (such as ED) or compared outcomes before and during SDEC implementation. Patients receiving SDEC demonstrated same-day discharge ranging from 38.3% to >92%. 30-day mortality varied between <1% and 6% (four studies). Change in 30-day ED or SDEC reattendance was not examined. A learning curve was indicated in two studies, with inappropriate or ‘rejected’ referrals reducing from 31% to 18% in one SDEC service. Where reported, triage was led by senior clinical decision-makers, however, the appropriateness of SDEC referrals was also complicated by contextual factors. Comparative evaluation, including inpatient admissions, generally favoured SDEC care, but study designs carried a high risk of bias and confounding.

**Conclusions:**

Limited evidence suggests that implementing SDEC services is feasible and may increase same-day discharge but with variable 30-day mortality (very-low or low confidence) and unexamined change in 30-day ED or SDEC reattendance. Clinical heterogeneity and limited reporting make it difficult to characterise SDEC services. Implementation, although with varied referral criteria, proved feasible given the involvement of senior clinical decision-makers.

**WHAT IS ALREADY KNOWN ON THIS TOPIC**

- Same day emergency care (SDEC) describes an ambulatory emergency care service designed to provide an alternative to ED management, reduce admission rates and improve emergency care system performance.

**WHAT THIS STUDY ADDS**

- SDEC services are feasible to implement and may increase same-day discharge but show variable 30-day mortality and unexamined change in 30-day ED or SDEC reattendance. Our confidence in the evidence for intervention effect, to date, remains low to very low.

**HOW THIS STUDY MIGHT AFFECT RESEARCH, PRACTICE OR POLICY**

- SDEC may offer a viable alternative to the ED, but further research is required to characterise SDEC services and determine their effect on emergency care system performance.

## Introduction

 ED crowding is an international problem characterised by prolonged waiting times and treatment delays that occur when demand for ED care outstrips supply or when ED outflow is limited by lack of available inpatient beds.^1^ Same day emergency care (SDEC), or ambulatory emergency care (AEC), is an innovative healthcare model, used predominantly in the UK. SDEC exists as an alternative specialist pathway for rapid referral of patients to the ED who may otherwise be admitted to an inpatient bed. SDEC aims to provide a targeted service for such selected patients, supporting timely discharge and reducing unnecessary inpatient admissions. It should also reduce some of the pressure on the ED by providing a specific emergency service for patients who are likely to be discharged within 12 hours and who, if necessary, can attend again on subsequent days for further investigation or review, rather than being admitted.^2 3^ SDECs typically operate for around 12 hours per day during daytime hours of peak ED demand and aim to complete assessment within an 8-hour timeframe to facilitate same-day discharge. Some SDECs offer next-day review for patients who would otherwise have been admitted overnight for urgent but not emergency investigations, and where other outpatient services are unavailable. If SDECs can reduce admissions, then this could reduce ED crowding due to patients awaiting admission (ED boarding) when there is a lack of inpatient beds (exit block). However, there is no universal definition for SDEC, and some variation may be expected between services led by different clinical specialties (eg, surgery or medicine). In England, the NHS strategy requires SDEC implementation at every hospital with a ‘type 1’ (consultant-led 24 hours) ED, with 75% of acute hospitals meeting this requirement in January 2024.^4^

Patients can be referred to SDEC through ED streaming or triage, or direct referral from primary care, ambulance services or telephone triage services. In England, clinical guidance on the selection of patients for SDEC is intended to ensure patient safety and the use of SDEC as a credible alternative to ED management and inpatient admission.^5^ However, no accepted, single set of criteria for referral currently exists, such that SDEC services are implemented in different ways in different hospitals, and for many different patient profiles.^2 3^ Services can therefore be acute medical or other specialised, and the training and composition of the SDEC team are undefined. NHS guidance does not currently identify disease-specific patient groups or sources of referral beyond an aspiration for hospitals to provide an acute clinical frailty assessment service within 30 min of arrival in the ED/SDEC unit.^5^ Appropriate patient selection may be crucial to ensuring that SDEC achieves the aim of reducing hospital admissions, as well as ED demand, and improving patient experience. Newly commissioned research includes plans to examine SDEC implementation across all type 1 EDs in England.^6^

Implementation of new services should be based on evidence of effectiveness. Scoping or narrative reviews of SDEC have been published,^3 7^ but no published systematic review evaluates whether SDEC improves emergency care system outcomes. We aimed to undertake a systematic review to determine the impact of SDEC on emergency care system outcomes, using evidence from different types of evaluation, including audit, service improvement and primary research.

## Methods

We conducted a systematic review following established guidelines^8^ and reported according to the Preferred Reporting Items for Systematic reviews and Meta-Analyses (PRISMA) checklist^9^ (online supplemental appendix 1). This work initially considered primary and secondary research as part of a broader project (PROSPERO, CRD: 42024502585) focusing on the 10 high-impact initiatives for urgent and emergency care, identified by NHS England, but examining the outcomes of waiting times and ambulance response times only.^7^

### Search strategy and eligibility criteria

An information specialist (AS) designed and conducted searches to identify relevant primary research and service evaluations. The searches combined free-text and available thesaurus terms for the intervention and setting across the following eight bibliographic databases: MEDLINE via Ovid; EMBASE via Ovid; PsycInfo via Ovid; CINAHL via EBSCO; the Science and Social Science Citation Indices in the Web of Science Core Collection; Health Management Information Consortium; the Cochrane Library; and Epistemonikos. These searches covered the period from January 2018, the date from which SDEC or equivalent terminology was first used. Included publications were restricted to English only. The full search strategies were conducted in February 2024 and are provided in online supplemental appendix 2.

Studies were included if they were an evaluation of a service clearly labelled as SDEC or AEC, and assessment included at least one outcome of interest: admission or discharge rates, referral rates or mortality.

### Study selection and data extraction

Screening of titles/abstracts and full texts was conducted independently by two reviewers (KJ and CC). Any citation identified as relevant by either reviewer was retrieved. All full-text items were also double screened (KJ/CC). Disagreements were resolved by consensus or reference to a subject expert if necessary (CM). Data items comprised first author, year, country/setting, sample size (n=patients), study design and duration, population details, intervention details, methods of data collection and analysis, findings for outcomes of interest. Data were extracted by one reviewer and checked by a second; disagreements were resolved by consensus.

### Implementation, quality assessment and synthesis

Two reviewers (KJ and CC) conducted an independent formal assessment of quality improvement with SDEC, including study limitations, using the Quality Improvement Minimum Quality Criteria Set (QI-MQCS), a validated tool for assessment of quality improvement evaluations in healthcare.^10^ This tool represented the best fit for assessing how SDEC was being delivered within an emerging evidence base. We would have performed a formal assessment for risk of bias (using Risk Of Bias In Non-randomized Studies—of Interventions) if the review evidence had used confirmatory rather than exploratory/preliminary research designs. We judged the risk of bias and our confidence in outcomes based on non-randomised study design limitations. Given the heterogeneity of populations, interventions (service variations), outcomes and outcome measurement, the evidence base and its findings are reported as a narrative synthesis.

## Results

**Figure 1:**
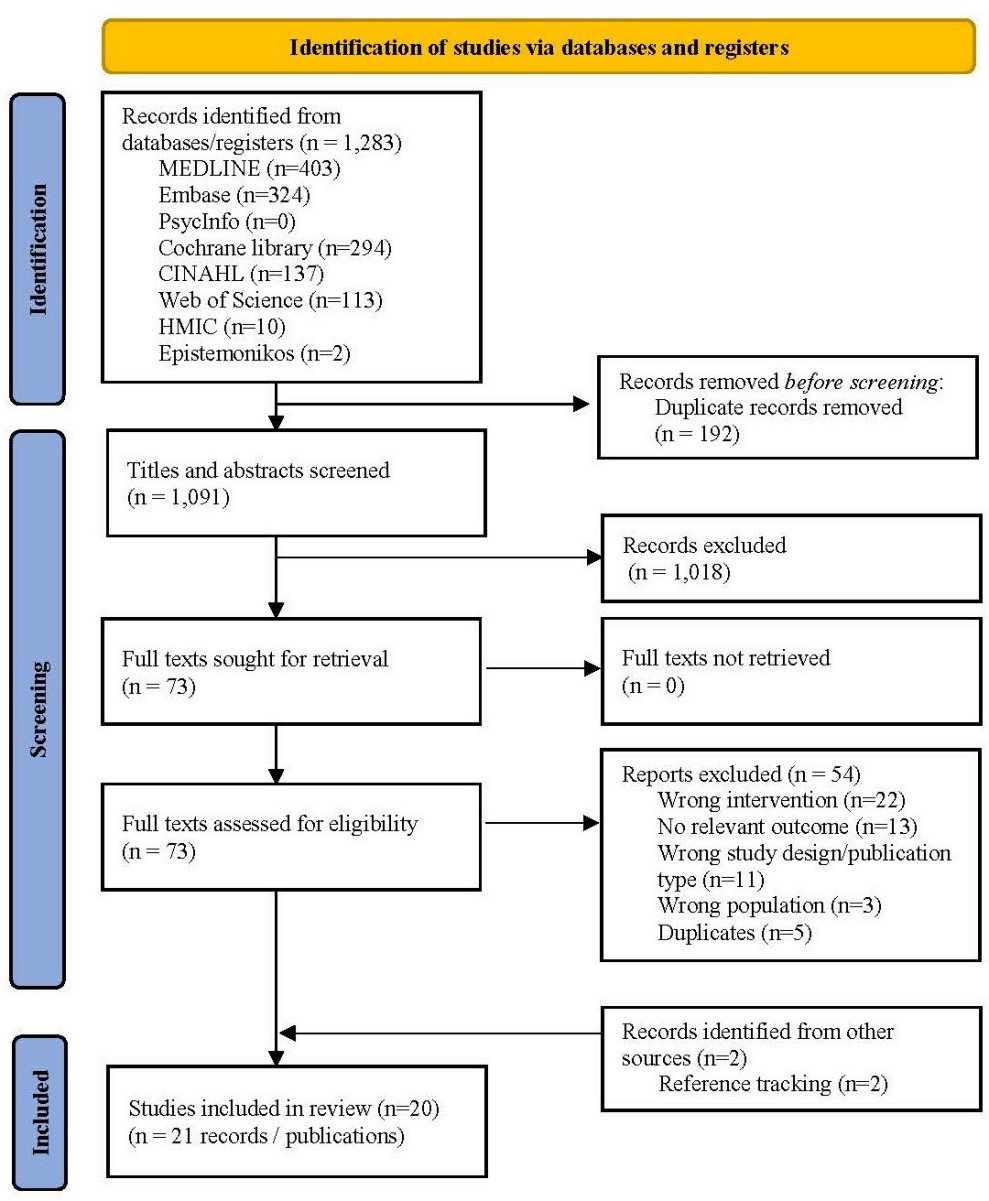
Flow diagram.

The search of bibliographic databases yielded a total of 1283 citations. After screening titles and abstracts, 73 publications were checked as full texts and 19 publications were found to satisfy the eligibility criteria. 54 full-text publications were excluded, principally for assessing an intervention that would not be considered SDEC, or only reporting a non-relevant outcome (eg, acceptability, length of stay). All reasons for exclusion are reported in online supplemental appendix 3. One additional publication^11^ was identified from a relevant scoping review,^3^ and a second from tracking references of included studies,^12^ resulting in 20 included studies (21 publications). One study has two publications,^12 13^ and two studies were conducted in the same SDEC.^11^ ^14^ The results of this literature search and screening process are summarised in the PRISMA flow diagram (figure 1).

Table 1 summarises the characteristics of included studies. Table 2 summarises the SDEC services of the included studies. We use the term SDEC to describe all services, but some studies used alternative terms eg, AEC^15^ ^16^ or they described acute/emergency ambulatory services eg, ambulatory cardiology unit.^17^ One SDEC was reported to form part of an acute medical unit (AMU)^14^ although this was a distinct service in other studies.^18 19^ All included studies were conducted in the UK, except for two conducted in Australia.^16 20^ Two studies were conducted across multiple centres and services^18 19^ and one in two sites within a single trust,^15^ but the majority (n=17) were single-centre evaluations. Where reported, the number of SDEC-attending patients analysed for review outcomes ranged from 24 (29% of 82 general practitioner (GP) referrals)^21^ to 16497 (mixed sources of referral)^22^ and the prospective data collection period ranged from 24 hours (in successive years)^18^ ^19^ to 38 months.^22^

**Table 1:** Study characteristics

First author, year	Country	Study design	Study duration	Sample size (n=patients unless otherwise stated)
Ali, 2018^28^	UK	Audit	30 days (1–30 October 2017)	n=260 attendances
Atkin, 2022^19^	UK	Comparative audit	24 hours in June 2021, 2019 and Winter 2020	n=158 units in 156 UK hospitals
Atkin, 2023^18^	UK	Comparative audit	24 hours in June 2022, 2019, 2020 and 2021	n=152 units in 149 UK hospitals (131 in England)
Baker, 2018^15^	UK	Before-and-after retrospective service review	Two consecutive years in Jul (2012–2014)	n=191 (2013); n=344 (2014)
Baker, 2019^21^	UK	Plan–do–act–study	2 weeks	n=24
Balaratnam, 2022^25^	UK	Non-comparative, service evaluation	From May 2021	n=approximately 100 referrals/month
Byrne, 2018^24^	UK	Before-and-after study	2 years (2014–2015)	n=946 (ACU new patients only)
Corvan, 2022^17^	UK	Preintervention and postintervention audit	3 months before/3 months after intervention	n=115 (suitable patients pre intervention); n=137 patients referred (post intervention)
Edison, 2021^23^	UK	Prospective cohort study with benchmark outcome	3 months	n=175
Elias, 2021^30^	UK	Prospective observational study	August–December 2015	n=533
Hsu, 2022^31^	UK	Service evaluation	March–August 2021	n=255
Jarral, 2020^26^	UK	Includes cross-sectional and comparative data	4 days in September 2019 and August 2019	n=33 (seen in SDEC)
Keane, 2022^16^	Australia	Quality improvement study	7 months (2 February to 31 August 2021)	n=5507
Keaney, 2019^14^	UK	Audit and reaudit	2 split weeks	n=206
Pincombe, 2023^20^	Australia	Economic evaluation with single group interrupted time series data	January 2016 to December 2020	n=2285 (1387 new and 898 reviews)
Ray, 2020,^13^ 2020^12^	UK	Audit and reaudit	Not reported	n=100
Reddy, 2022^27^	UK	Before-and-after service evaluation	Not reported	Prepathway n=167; postpathway n=139
Reschen, 2020^22^	UK	Retrospective, comparative study	38 months (December 2015 to March 2019)	n=16 497
Visanji, 2020^11^	UK	Before-and-after quality improvement study	Unclear comparison time periods	Not reported
Weihser, 2018^29^	UK	Two-phase quality improvement study with before-and-after comparison	15 months (including initial 3-month pilot)	n=422 (pilot); n=501 (mean per month over 1 year)

ACU, ambulatory cardiology unit; SDEC, same day emergency care.

**Table 2:** SDEC characteristics

First author, year	Type(s) of service model	SDEC selection criteria	Source of referrals
Ali, 2018^28^	‘Ambulatory care service’ (GM/specialities not specified)	‘AMBS criteria’ and mixed ambulatory care models	Unclear although audit findings were fed back to primary care colleagues
Atkin, 2022^19^	'AEC/SDEC' versus AMU versus ED for unplanned medical attendances to acute medicine. ‘Frailty units accepting unplanned admissions’ were also included (acute medical)	Not reported	Directly from paramedics and indirectly via telephone triage of GP referrals (by consultants, registrars, core trainees, ANPs, nurses, ACPs or administrative staff)
Atkin, 2023^18^	SDEC versus AMU versus ED for unplanned medical attendances to acute and general internal medicine (acute medical)	Not reported	ED and primary care*
Baker, 2018^15^	SDEC ('AEC') service (GM/specialities not specified)	Based on SDEC local referral guidelines in use at the time, that is, the AMB Score, for predicting the likelihood of same-day discharge	Not reported
Baker, 2019^21^	ACP added to existing surgical SDEC ('AEC')	Not reported (‘strict criteria’ with streaming of patients from ED to SDEC for assessment)	GP
Balaratnam, 2022^25^	Neurology SDEC (N-SDEC) service for acute neurology population embedded within ED	Not reported	Not reported
Byrne, 2018^24^	SDEC in form of an 'ACU'	‘conditions initially identified as suitable…included atrial fibrillation (AF) and other atrial arrhythmias, new or worsening heart failure, and syncope thought to have a cardiac cause’	ED (n=722); inpatient stream (n=40); urgent outpatient stream (n=122); clinics (n=51); not reported (n=11)
Corvan, 2022^17^	SDEC in form of an 'ACU'	Not reported	ED
Edison, 2021^23^	An emergency general surgery AEC/SDEC pathway	‘NEWS Score<4; able to wait/sit on chair; no acute confusion; no social issues; not clearly requiring a hospital admission; no infection control issues; agreed by senior decision-maker to be appropriate’	ED, urgent care centre or their GP, or as part of early facilitated discharge from an inpatient admission
Elias, 2021^30^	‘Specialist-led community hospital-based SDEC unit with multidisciplinary team input and inclusive referral criteria, termed EMU’ (GM/specialities not specified)	No absolute exclusion criteria	Primary care physicians, paramedics or local acute inpatient services (patients cannot self-present to the service)
Hsu, 2022^31^	SDEC ‘hot clinic’ running Monday–Friday (mostly neurology)	Not reported, but principally neurology	ED, from the Western Eye Hospital, local ophthalmology ED
Jarral, 2020^26^	‘Ambulatory care’ service/SDEC (GM/specialities not specified)	Not reported, but common conditions for example, chest pain/shortness of breath, palpitations, headache	ED only
Keane, 2022^16^	AEC Centre (SDEC) (GM/specialities not specified)	Australasian Triage Score of 3, 4 or 5 assessed using the modified AMB Score; patients scoring an AMBS>5 were streamed to the SDEC (conditional on exclusion criteria)	ED only
Keaney, 2019^14^	SDEC ('AEC') formed part of the AMU, using objectives and aims of NHS Improvement SDEC (GM/specialities not specified). This service involved an additional senior decision-maker	‘Fit to sit’	‘Acute medical’ on-call team, ED, GPs and inpatient wards (post discharge)
Pincombe, 2023^20^	'Medical ambulatory care' service for out-of-hospital care, including diagnostics and therapeutics for patients requiring urgent attention, but which can be safely administered in the ambulatory environment (GM/specialities not specified)	Not reported	GP, ED and ward
Ray, 2020,^13^ 2020^12^	SDEC ('AEC') surgical unit. Surgical patients. Pathway ‘delivered by a senior clinician’	Unclear	ED, GP and wards
Reddy, 2022^27^	‘In 2019, a front-door ED pathway…was created to direct low-risk chest pain towards ambulatory care’ (cardiology)	ED cohort who presented with ‘low risk’ cardiac chest pain	ED
Reschen, 2020^22^	'AEC'/SDEC pathway compared with standard, non-ambulatory pathway (EAU) (GM/specialities not specified)	Unclear	ED only for direct referral to the SDEC pathway and ED, GP/AHP, paramedics and ‘other’ for initial phone triage by SDEC consultant or resident medical officer ‘under direct consultant supervision’
Visanji, 2020^11^	New AMU, using ‘the objectives and aims set out by NHS Improvement’ SDEC (GM/specialities not specified)	‘Fit to sit’—focused on ‘stable patients’	GP (with streaming of patients through the ED)
Weihser, 2018^29^	Baseline and redesigned 'ambulatory care' service model in acute medicine pathway (SDEC) with point-of-care testing diagnostics (GM/specialities not specified)	Condition-specific patient management algorithms	Not reported

* These are the only reported sources of referral (table 1), but the sum is not 100%.

ACP, advanced clinical practitioner; AEC, ambulatory emergency care; AHP, allied health professional; AMBS, Ambulatory Multidisciplinary Assessment and Referral System; AMU, acute medical unit; ANP, advanced nurse practitioner; EAU, emergency assessment unit; EMU, emergency multidisciplinary unit; GM, general medicine; GP, general practitioner; SDEC, same day emergency care.

The SDEC services were heterogeneous. Patient selection, where described, often involved unplanned medical cases with a variety of specified criteria, but two studies (three publications) evaluated surgical SDEC services,^12 13 23^ two a cardiology service^17 24^ and one an acute neurology service.^25^ Referral sources varied from multiple sources, including the ED, primary care, paramedics and inpatient services, to just the ED in four studies.^16 17 26 27^ Four of the 20 evaluations did not specify the source of SDEC referrals.^15 25 28 29^ Where reported, the SDEC service was led by a consultant or other senior clinical decision-maker.

Study designs were cross-sectional,^13 16 18 19 21 23 25 26 28 30 31^ before-and-after comparisons,^11 15 17 24 27 29^ or other within-service comparisons^12 14 22^ - one was an economic evaluation with interrupted time series data.^20^ Cross-sectional studies included comparisons that were explicit or possible against benchmark standards, for example, target times to be seen^16 18 19^ or alternative service models, for example, the ED or an AMU.^18 19^ Review outcomes reported included daily to annual hospital admissions (13 studies^1215^,^18 22^) and overall or same-day hospital discharge (12 studies^1215^,^19 21 23 26 28 29 31^). One study reported completed care within one patient visit or visits over two consecutive days, and open access to return within 72 hours.^22^ Another study reported patient did not attend (DNA) within discharge outcomes.^23^ Other time-based outcomes reported were mortality (14 days^18^, 30 days^22 24 29 30^ or longer follow-up^22 30^), <48 hours to 30-day reattendance^16 19 24^ (unscheduled at 7 days^19^), conversion to admission (annual average^22 24 29^) and cost (30 days to 1 year).^17 20 24^ Data reporting also included SDEC referral and (in)appropriateness (%), onward referral (%), bed occupancy (mean overnight patients) and saved bed days (based on change in 1–3-day admissions or unreported calculation) (see online supplemental appendix 4). ED wait times/length of stay are reported separately in a previous scoping review.^7^ Nine studies were reported as conference abstracts only and were included in the context of an emerging evidence base for SDEC services.^1114 17 21 25^,^28 31^

### Implementation and quality assessment

**Figure 2:**
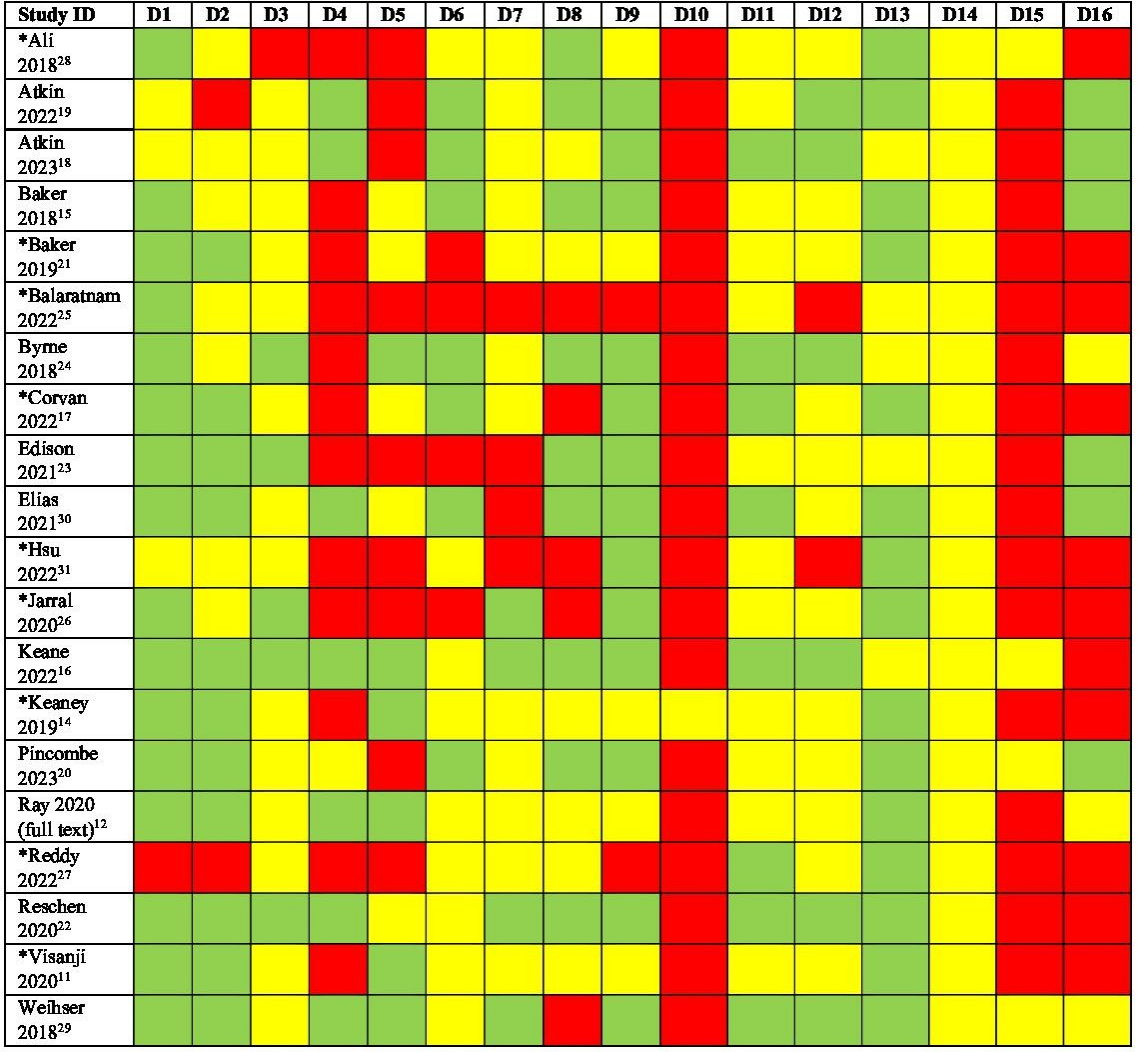
Quality assessment with the Quality Improvement Minimum Quality Criteria Set (QI-MQCS, V.1.0). *Abstract only. Green: dual assessment as quality criteria met; yellow: single assessment as quality criteria met or unclear; red: dual assessment as quality criteria not met QI-MQCS domains (extracted from Hempel *et al*^10^). D1: Organisational motivation (organisational problem, reason or motivation for the intervention). D2: Intervention rationale (rationale linking the intervention to its expected effects). D3: Intervention description (change in organisational or provider behaviour D4: Organisational characteristics (demographics or basic characteristics of the organisation). D5: Implementation (temporary activities used to introduce potentially enduring changes). D6: Study design (study design and comparator). D7: Comparator (information about comparator care processes). D8: Data source (data source and outcome definition). D9: Timing (timing of intervention and evaluation). D10: Adherence/fidelity (adherence to the intervention). D11: Health outcomes (patient health-related outcomes). D12: Organisational readiness (barriers and facilitators to readiness). D13: Penetration/reach (penetration/reach of the intervention). D14: Sustainability (sustainability of the intervention). D15: Spread (ability to be spread or replicated). D16: Limitations (interpretation of the evaluation).

Most studies contained a limited description of the intervention (D3) and organisational characteristics (D4) (figure 2). Adherence to the intervention was only addressed by one fidelity study^14^ (D10), and sustainability of SDEC (D14) was unclear. Overall, evidence of spread or failure to spread was lacking (D15). More than half of the included studies did not discuss limitations of the findings (D16).

Our confidence in the quantitative findings for review outcomes was low to very low. This was based on non-randomised study design limitations, including low internal validity to determine intervention effect and an increased risk of publication bias associated with preliminary research in the early-phase development of interventions.^32 33^

### Findings

Online supplemental tables 3 and 4 in appendix 4 summarise review and further study outcomes reported. Studies generally reported high overall discharge, while same-day discharge ranged from 38.3%^23^ to >92%.^26 28^ One before–after study involving point-of-care testing diagnostics found same-day discharges increased by 8.2% with an SDEC pilot.^29^ Studies comparing SDEC to ED discharge suggested that SDEC overall discharge was higher,^15 18^ although same-day discharge was not specified in one of them^15^. Also, these studies compared the proportions of SDEC discharges (i.e. not a true rate) using convenience sampling^15^ or against the combined proportional discharge from SDEC, AMU and ED services.^18^ Other studies compared inpatient admission before and during SDEC implementation to suggest that the SDEC service reduced admissions.^12 16 17 22 24 29^

Patients receiving the SDEC service demonstrated variable 30-day mortality (four studies), ranging from<1% (with ACU referral criteria)^24^ to 6% (primarily inpatients aged>65 years and with 'inclusive' EMU referral criteria - no exclusion criteria for patients assessed).^30^ We did not attempt to pool these data because of the clinical variation in patient eligibility. Mortality comparisons between SDEC and ED (0% vs 4.5% at 14 days),^18^ SDEC and emergency assessment unit (1.6% vs 8% at 30 days)^22^ and before versus after pilot and expanded SDEC implementation (4.5% vs 0.7% at 30 days)^29^ suggested lower mortality with SDEC care although the SDEC managed different populations in these comparisons from early-phase studies.

There was a lack of comparable evidence for the impact of SDEC services on ED or SDEC reattendance at 30 days. Eight studies reported details of the volume or appropriateness of referrals into the SDEC, with evidence from two studies of increasing referrals over time (from GPs via ED streaming or from direct ED referral and other sources triaged by a senior clinical decision-maker)^11 22^ and increased appropriate referrals from different sources in one study (38% to 54%).^12^ (In)appropriateness of referrals was assessed both among those managed in SDEC, and those not managed in SDEC,^12 23 26 28^ suggesting a mix of appropriate SDEC referral, 'missed opportunity' (under-utilised service), 'waste' (inappropriate, non-urgent cases) and 'clinical risk' (inappropriate cases requiring ED/admission).^12^ Two studies, respectively, reported that 5.1%^23^ and 18 to 22%^12^ of SDEC referrals were clinically inappropriate and these were non-urgent cases (attributed, in part, to the impact of the COVID-19 pandemic lockdown during re-audit),^12^ or required admission.^12 23^ Meanwhile, one of the studies identified an unchanged 6% of cases as ‘missed opportunities’ (ie, optimally managed in SDEC but managed in the ED instead).^12^ A clear learning curve was indicated in two studies, with inappropriate or ‘rejected’ referrals reducing from 31% to 18% in one service,^14^ while the proportion of GP-referred/ED streamed patients seen in SDEC increased from 9% to 47% in another service.^11^ A single retrospective study of SDEC found that bed occupancy remained consistent over 3 years despite increased volumes of patients seen through this service.^22^ Saved bed days were reported in two other before-and-after evaluations.^17 29^

## Discussion

Our systematic review identified a developing evidence base evaluating SDEC services, currently limited by the clinical heterogeneity of services and weak study designs for determining intervention effect. SDEC services varied in their sources of referral, specialties provided and patient selection criteria, with many studies providing limited description of the SDEC. We found most studies missed contextual information on the SDEC specialty and team composition, which may have confounded review outcomes. Descriptive outcomes suggested that SDEC services were feasible, associated with low admission and high overall discharge. However, only one study involving point-of-care testing diagnostics showed improved same-day discharge, and overall discharge may have included patient DNA. Reporting of referrals suggested an increase and that referrals may become more appropriate over time but needs to consider various contextual factors for implementation (eg, adherence to intervention and sustainability). As reported elsewhere,^7^ studies included here have also mostly reported a reduction in ED waiting times following SDEC implementation or that patients managed by the SDEC experienced shorter waiting times.^11 26 29^ However, one study reported increased stay time, related to increased workload.^12^ It remains uncertain whether SDEC services had a significant impact on ED and SDEC reattendances based on the current review, with scheduled follow-up embedded in some SDEC services. Five studies included in this review reported economic data alongside outcomes of interest; one estimated increased cost if increased ED attendances had all been associated with one-night admissions,^24^ but the rest reported some form of cost saving (including bed days saved) due to SDEC.^17 20 23 29^

The majority of evaluations of effectiveness were limited to comparing outcomes to other services (although SDEC and ED discharge are likely to reflect different populations, representing selection bias) or comparing outcomes before versus after SDEC implementation (such ‘before vs after’ studies provide poor evidence of effectiveness^34 35^). These designs carry a high risk of bias and confounding. The cost-effectiveness of the service also needs to be evaluated more robustly than has been done to date. In the absence of sufficiently robust, comparative study designs and reporting, we considered our confidence in the evidence to be low or very low across review outcomes. We are therefore unable to draw definitive conclusions at this time regarding the effect of SDEC services on broader outcomes including admission rates and saved bed days among other indicators of emergency care system performance.

A scoping review of studies of adult medical SDEC services previously identified two observational cohort studies and four audits (all included in this review), and concluded that this scarcity of the literature highlights a need for further study.^3^ Findings from another review of a wider variety of high-impact initiatives identify some evidence that SDEC, acute frailty units, care transfer hubs and some inpatient flow interventions might reduce ED waiting times.^7^ We here report our findings and conclusions related to SDEC services. Given the heterogeneity of these services, there might be some shared potential implications for triage and implementation that are applicable across other service models, such as acute frailty units.

Our systematic review has strengths and limitations. We followed established guidelines^8^ and used a comprehensive and inclusive search strategy to identify all the relevant literature. We may have excluded potentially eligible studies if they used an unfamiliar term to describe an SDEC service. We based our definition of SDEC on UK sources,^4 5^ and so may have missed studies from other countries of similar services using different terms. However, we tried to mitigate the potential impact of diverse terminology through approaches such as reference tracking, which enabled us to identify two additional publications. Given the challenge of identifying a single appraisal tool for early-phase research with heterogeneous study designs, our choice of the QI-MQCS tool was a pragmatic one. The implementation quality assessment was conducted by two reviewers independently, with discrepancies across some domains. Aside from potential issues with the application of the tool, such discrepancies emphasise a need for caution when interpreting judgements on quality improvement. Nevertheless, the QI-MQCS tool helped to identify a range of implementation factors for the evaluation of SDEC services, looking beyond outcome metrics.

We included any type of service evaluation and conference abstracts to capture the emerging evidence on SDEC services. However, this available evidence yielded limited information with a high risk of selection bias. Our conclusions are inevitably limited by the primary data, particularly the limited reporting of SDEC characteristics, clinical heterogeneity of the SDEC services and study designs that do not support sufficiently reliable evaluation of effect.

Despite the limitations of the available literature, we can identify some policy and practice implications. Descriptive data suggest that SDEC services are feasible and, for some patients, may increase same-day or subsequent discharge although more research is needed to establish if this is a viable alternative to ED and inpatient care. SDEC services are framed in the NHS England Urgent and Emergency Care recovery plan as one of ten high-impact initiatives aimed at reducing ED wait times. However, later policy documents, including the Royal College of Emergency Medicine/Society of Acute Medicine position statement, stress the role of SDEC services in reducing inpatient admissions and that they should be used only for patients considered for inpatient admission. Providing patients with quicker specialist review and specialist investigations might also enhance patient outcomes for these services. Analysis of referrals suggests that a learning curve may need to be negotiated before their full potential is realised. Included studies describe SDEC services that are led by consultants or other, senior clinical decision-makers, that involve a variety of different specialties and that manage a variety of presenting complaints. The heterogeneity of services described and lack of reliable effectiveness data mean that we are unable to identify a reproducible SDEC model or reliably predict whether SDEC implementation will improve emergency care outcomes.

Further research is required to guide policy and practice around SDEC implementation. Fuller descriptions in both quantitative and qualitative studies are needed to characterise SDEC models and develop a clear definition of what SDEC does (and does not) involve. Multicentre mixed methods evaluation could further determine whether SDEC improves emergency care system performance, identify those SDEC models that are most likely to be effective and determine facilitators and barriers to high-quality SDEC care.

## Conclusion

The available literature suggests that SDEC may provide an alternative to the ED for some patients. However, there is currently limited evidence for improved same-day (and overall) discharge, how this relates to inpatient admissions, ED or SDEC reattendance (and DNA), referral rates and mortality. Insufficient reporting and clinical heterogeneity in the services as described make it difficult to characterise exactly what an SDEC involves. Our confidence in the evidence for urgent and emergency care delivery outcomes, to date, is low to very low, with limited comparative evaluation to determine the effect of SDEC services.

## Supplementary material

10.1136/emermed-2024-214821 online supplemental file 1

10.1136/emermed-2024-214821 online supplemental file 2

## Data Availability

All data relevant to the study are included in the article or uploaded as online supplemental information.

## References

[1] Pines JM, Hilton JA, Weber EJ (2011). International perspectives on emergency department crowding. Acad Emerg Med.

[2] Atkin C, Gallier S, Wallin E (2022). Performance of scoring systems in selecting short stay medical admissions suitable for assessment in same day emergency care: an analysis of diagnostic accuracy in a UK hospital setting. BMJ Open.

[3] Dean S, Barratt J (2024). What is the existing evidence base for adult medical Same Day Emergency Care in UK NHS hospitals? A scoping review. Future Healthc J.

[4] RCEM S (2024). Joint statement RCEM and SAM regarding same day emergency care (SDEC). https://rcem.ac.uk/joint-statement-rcem-and-sam-regarding-same-day-emergency-care-sdec.

[5] NHS England (2024). SAMEDAY strategy – a framework for the development and delivery of same day emergency care.

[6] Dean S (2025). What are the clinical outcomes and patient experiences of adults accessing medical same day emergency care services? a multi-phase design mixed methods study. (The SDEC study). https://fundingawards.nihr.ac.uk/award/NIHR304566.

[7] Carroll C, Kundakci B, Muhinyi A (2024). Scoping review of the effectiveness of 10 high-impact initiatives (HIIs) for recovering urgent and emergency care services. *BMJ Open Qual*.

[8] Centre for Reviews and Dissemination (CRD), University of York (2008). Systematic Reviews. CRD’s Guidance for Undertaking Reviews in Health Care.

[9] Page MJ, McKenzie JE, Bossuyt PM (2021). The PRISMA 2020 statement: an updated guideline for reporting systematic reviews. BMJ.

[10] Hempel S, Shekelle PG, Liu JL (2015). Development of the Quality Improvement Minimum Quality Criteria Set (QI-MQCS): a tool for critical appraisal of quality improvement intervention publications. *BMJ Qual Saf*.

[11] Visanji S, Lyne H, Phillips A (2020). Using the principles of “same day emergency care” in our new acute medical unit. Future Healthc J.

[12] Ray K, Mittal A, Swaminathan C (2020). The Use of Ambulatory Emergency Care Unit as Supply-Side Drivers for Non-Emergency Surgical Patients during COVID-19 Pandemic Lockdown: An Audit Report from a Tertiary Care Center. Clin Surg J.

[13] Ray K, Mittal A, Singh K (2020). A retrospective study evaluating the performance of an Ambulatory Emergency Care Unit for Surgical Patients in a tertiary care NHS hospital. ASGBI Abstracts.

[14] Keaney K, Sweeney A, Visanji S (2019). Improving the quality of referrals to ambulatory emergency care. Future Healthc J.

[15] Baker J (2018). Effects of ambulatory emergency care on organisational and patient outcomes. Nurs Manag (Harrow).

[16] Keane C, Clayden V, Scott G (2022). Evaluation of an Ambulatory Emergency Care Centre at a tertiary hospital in Perth, Western Australia. Australas Emerg Care.

[17] Corvan F, McKane M, McGrath P (2022). Ambulatory cardiology unit – an innovative, safe, high quality service, reducing hospital admissions. Heart.

[18] Atkin C, Knight T, Cooksley T (2023). Performance of admission pathways within acute medicine services: Analysis from the Society for Acute Medicine Benchmarking Audit 2022 and comparison with performance 2019 - 2021. Eur J Intern Med.

[19] Atkin C, Knight T, Cooksley T (2022). Society for Acute Medicine Benchmarking Audit 2021 (SAMBA21): assessing national performance of acute medicine services. Acute Med.

[20] Pincombe A, Schultz TJ, Hofmann D (2023). Economic evaluation of a medical ambulatory care service using a single group interrupted time-series design. J Eval Clin Pract.

[21] Baker J, Krishnamoorthy A, Busb K (2019). Could the use of an Advanced Clinical Practitioner Led Ambulatory Emergency Care Unit improve flow in Emergency Surgery?. BJS.

[22] Reschen ME, Bowen J, Singh S (2020). Process of care and activity in a clinically inclusive ambulatory emergency care unit: progressive effect over time on clinical outcomes and acute medical admissions. Future Healthc J.

[23] Edison MA, Waskett B, Adekitan D (2021). Clinical benefits of a combined physician associate and senior specialist-led emergency surgery ambulatory emergency care clinic introduced in response to the COVID-19 pandemic. *BMJ Open Qual*.

[24] Byrne J, McCall D (2018). One year’s activity and outcome data from an ambulatory cardiology unit. Clin Med (Lond).

[25] Balaratnam M, Alim-Marvasti A-J, Lane C (2022). Delivering acute neurology care via the Same Day Emergency Care (SDEC) model. J Neurol Neurosurg Psychiatry.

[26] Jarral W, Graf B, Harding S (2020). Direct referrals from emergency department streaming to ambulatory care: improving same-day emergency care. Future Healthc J.

[27] Reddy RK, Wang BX, Gwyther B (2022). Abstract 15713: A Novel Chest Pain Pathway Triaging Low-Risk Emergency Department Patients via Ambulatory Care Reduces Hospital Admissions Without Increased 30-Day Readmissions or All-Cause Mortality. Circulation.

[28] Ali A, Karmani J (2018). Audit of a newly developed ambulatory care service at Diana Princess of Wales Hospital Grimsby UK. Postgrad Med J.

[29] Weihser P, Giles D (2018). Establishing an ambulatory care service using point-of-care testing diagnostics. Br J Hosp Med (Lond).

[30] Elias TCN, Bowen J, Hassanzadeh R (2021). Factors associated with admission to bed-based care: observational prospective cohort study in a multidisciplinary same day emergency care unit (SDEC). BMC Geriatr.

[31] Hsu C, Elrhermoul F-Z, Maduakor C (2022). 015 Service evaluation: the role of same day emergency care in managing acute neurological presentations. J Neurol Neurosurg Psychiatry.

[32] Malmivaara A (2015). Methodological considerations of the GRADE method. Ann Med.

[33] von Klinggraeff L, Ramey K, Pfledderer CD (2023). The mysterious case of the disappearing pilot study: a review of publication bias in preliminary behavioral interventions presented at health behavior conferences. Pilot Feasibility Stud.

[34] Goodacre S (2015). Uncontrolled before-after studies: discouraged by Cochrane and the EMJ. *Emerg Med J*.

[35] Lijmer JG, Mol BW, Heisterkamp S (1999). Empirical evidence of design-related bias in studies of diagnostic tests. JAMA.

