# Supplementary material for: Impact of same day emergency care services on urgent and emergency care delivery outcomes: a systematic review
Source: Emerg Med J. 2025 Jul 7;42(10):e214821. doi: 10.1136/emermed-2024-214821 (PMC12505097; doi:10.1136/emermed-2024-214821)
Supplement: online supplemental file 1 [file emermed-42-10-s001.pdf]

Appendix 2 Search strategies

Ovid MEDLINE(R) and Epub Ahead of Print, In-Process, In-Data-Review & Other Non-Indexed Citations and Daily <1946 to February 12, 2024>

1	(same day emergency care or same-day emergency care).mp.	28
2	SDEC.ti,ab.	85
3	*Ambulatory Care/	20860
4	*Emergency Service, Hospital/	47543
5	*Emergency Medical Services/	36399
6	*Emergency Medicine/	11957
7	(emergency adj2 service*).ti,ab.	22063
8	"emergency care".ti,ab.	11778
9	"urgent care".ti,ab.	3363
10	"emergency department* ".ti,ab.	130816
11	"accident and emergency".ti,ab.	5049
12	casualty.ti,ab.	6807
13	or/4-12	210650
14	1 or 2	99
15	3 and 13	1284
16	ambulatory emergency care.ti,ab.	43
17	15 or 16	1316
18	AEC.ti,ab.	2625
19	13 and 18	30
20	14 or 17 or 19	1331
21	limit 20 to yr="2018 -Current"	403

Embase <1974 to 2024 Week 06>

1	(same day emergency care or same-day emergency care).mp.	46
2	SDEC.ti,ab.	128
3	*ambulatory care/	13994
4	*hospital emergency service/	2462
5	*emergency health service/	52636
6	*emergency medicine/	30056
7	(emergency adj2 service*).ti,ab.	30586
8	"emergency care".ti,ab.	15612
9	"urgent care".ti,ab.	5599
10	"emergency department* ".ti,ab.	201197
11	"accident and emergency".ti,ab.	6652
12	casualty.ti,ab.	8105
13	or/4-12	300811
14	1 or 2	143
15	3 and 13	804
16	ambulatory emergency care.ti,ab.	74
17	15 or 16	865
18	AEC.ti,ab.	4733
19	13 and 18	63
20	14 or 17 or 19	899
21	limit 20 to yr="2018 -Current"	324

APA PsycInfo <1806 to February Week 2 2024>

1	(same day emergency care or same-day emergency care).mp.	0
2	SDEC.ti,ab.	3

3	ambulatory emergency care.ti,ab.	0
4	AEC.ti,ab.	110
5	1 or 2 or 3 or 4	113
6	(emergency adj2 service*).ti,ab.	3473
7	"emergency care".ti,ab.	1082
8	"urgent care".ti,ab.	414
9	"emergency department* ".ti,ab.	11531
10	"accident and emergency".ti,ab.	517
11	casualty.ti,ab.	844
12	or/6-11	16340
13	5 and 12	0

CINAHL Tuesday, February 13, 2024 3:52:34 PM

S1: TI ((same day emergency care OR same-day emergency care)) OR AB ((same day emergency care OR same-day emergency care)) (27 results)

S2: TI SDEC OR AB SDEC (17 results)

S3: TI ambulatory emergency care OR AB ambulatory emergency care (342 results)

S4: TI AEC OR AB AEC (415 results)

S5: (S1 OR S2 OR S3 OR S4) (781 results)

S6: (MM "Ambulatory Care") (7,328 results)

S7: (MM "Emergency Service") OR (MM "Emergency Medical Services") OR (MM "Emergency Services, Psychiatric") OR (MM "Emergency Room Visits") (58,354 results)

S8: (MM "Emergency Medicine") (6,583 results)

S9: TI (emergency N2 service*) OR AB (emergency N2 service*) (13,178 results)

S10: TI "emergency care" OR AB "emergency care" (7,344 results)

S11: TI "urgent care" OR AB "urgent care" (2,205 results)

S12: TI "emergency department* " OR AB "emergency department* " (73,005 results)

S13: TI ("accident and emergency") OR AB ("accident and emergency") (2,470 results)

S14: TI casualty OR AB casualty (4,568 results)

S15: (S6 OR S7 OR S8 OR S9 OR S10 OR S11 OR S12 OR S13 OR S14) (132,676 results)

S16: (S1 OR S2) (40 results)

S17: (S3 AND S15) (303 results)

S18: TI ambulatory emergency care OR AB ambulatory emergency care (342 results)

S19: (S17 OR S18) (342 results)

S20: TI AEC OR AB AEC (415 results)

S21: (S15 AND S20) (23 results)

S22: (S19 OR S21) (350 results)

S23: (S19 OR S21) Limiters - Publication Date: 20180101-20241231

(137 results)

Web of Science Core Collection (Science Citation Index, Social Sciences Citation Index)

#1 "same day emergency care" or "same-day emergency care" (Topic) 25
#2 SDEC (Title) or SDEC (Abstract) 129
#3 (TI=(emergency NEAR/2 service* OR "emergency care" OR "urgent care" OR "emergency department*" OR "accident and emergency" OR casualty)) OR AB=(emergency NEAR/2 service* OR "emergency care" OR "urgent care" OR "emergency department*" OR "accident and emergency" OR casualty) 175,897
#4 (TI=(AEC)) OR AB=(AEC) 7,353
#5 #3 AND #4 34
#6 TI=("ambulatory emergency care") OR AB=("ambulatory emergency care") 34
#7 #6 AND #3 34
#1 OR #2 OR #5 OR #7 and 2018 or 2019 or 2020 or 2021 or 2022 or 2023 or 2024 (Publication Years) and Science Citation Index Expanded (SCI-EXPANDED) or Social Sciences Citation Index (SSCI) (Web of Science Index) 113

HMIC Health Management Information Consortium <1979 to November 2023>

1	(same day emergency care or same-day emergency care).mp.	7
2	SDEC.ti,ab.	3
3	Ambulatory care/	255
4	Urgent care/	38
5	(emergency adj2 service*).ti,ab.	1101
6	"emergency care".ti,ab.	702
7	"urgent care".ti,ab.	227
8	"emergency department* ".ti,ab.	2222
9	"accident and emergency".ti,ab.	1791
10	casualty.ti,ab.	334
11	or/4-10	4645
12	1 or 2	7
13	3 and 11	28
14	ambulatory emergency care.ti,ab.	23
15	13 or 14	32
16	AEC.ti,ab.	18
17	12 or 15 or 16	36
18	limit 17 to yr="2018 -Current"	10

CDSR/CENTRAL

Date Run: 15/02/2024 23:01:13

ID	Search	Hits
#1	(same day emergency care or same-day emergency care):ti,ab	394
#2	SDEC:ti,ab	9
#3	MeSH descriptor: [Ambulatory Care] this term only	3896
#4	MeSH descriptor: [Emergency Service, Hospital] this term only	3632
#5	MeSH descriptor: [Emergency Medical Services] this term only	1479
#6	MeSH descriptor: [Emergency Medicine] this term only	363
#7	(emergency NEAR/2 service*):ti,ab,kw	7278
#8	("emergency care" or "urgent care" or emergency department* or "accident and emergency" or casualty):ti,ab,kw	16678

#9	#4 or #5 or #6 or #7 or #8	20005
#10	#3 AND #9	163
#11	ambulatory emergency care:ti,ab	685
#12	#10 #11	109
#13	AEC:ti,ab	4243
#14	#13 and #9	38
#15	#12 OR #14 with Cochrane Library publication date Between Jan 2018 and Feb 2024	73
#16	#1 OR #2	403
#17	#16 AND #9	251
#18	#15 OR #17 with Cochrane Library publication date Between Jan 2018 and Feb 2024	294

Epistemonikos

(title:("same day emergency care") OR abstract:("same day emergency care")) OR (title:("same-day emergency care") OR abstract:("same-day emergency care")) OR (title:("ambulatory emergency care") OR abstract:("ambulatory emergency care"))
N=2

Appendix 3 Excluded studies with reasons (more than one assigned reason might apply to a publication)

Not specifically evaluating a SDEC intervention related to the ED (n=22)

1. Aghajafari F, Sayed S, Emami N, Lang E, Abraham J. Optimizing emergency department care transitions to outpatient settings: A systematic review and meta-analysis. American Journal of Emergency Medicine. 2020;38(12):2667-80.
2. Atkin C, Gallier S, Wallin E, Reddy-Kolanu V, Sapey E. Performance of scoring systems in selecting short stay medical admissions suitable for assessment in same day emergency care: an analysis of diagnostic accuracy in a UK hospital setting. Bmj Open. 2022;12(12).
3. Barkett NL, Weiss G, High B, Kurtin SE. Implementation of an Oral Antineoplastic Therapy Program: Results From a Pilot Project. Clinical Journal of Oncology Nursing. 2022;26(1):61-70.
4. Benjamin P, Bryce R, Oyedokun T, Stempien J. Strength in the gap: A rapid review of principles and practices for urgent care centres. Healthcare Management Forum. 2023;36(2):101-6.
5. Berthelot S, Mallet M, Blais S, Moore L, Guertin JR, Boulet J, et al. Adaptation of time-driven activity-based costing to the evaluation of the efficiency of ambulatory care provided in the emergency department. JACEP Open. 2022;3(4) (no pagination).
6. Bibok MB, Votova K, Balshaw RF, Lesperance ML, Croteau NS, Trivedi A, et al. Reducing time-to-unit among patients referred to an outpatient stroke assessment unit with a novel triage process: a prospective cohort study. BMC Health Services Research. 2018;18(1):142.
7. Bou Malham C, El Khatib S, Cestac P, Andrieu S, Rouch L, Salameh P. Impact of pharmacist-led interventions on patient care in ambulatory care settings: A systematic review. International Journal of Clinical Practice. 2021;75(11):e14864.
8. Bunn JG, Croft SJ, O'Keeffe C, Jacques RM, Simpson RM, Stone T, et al. Urgent care axis for the older adult: where is best to target interventions? Emergency Medicine Journal. 2019;36(1):22-6.
9. Chalk D. Using computer simulation to model the expansion needs of the ambulatory emergency care unit at Derriford Hospital. Future Healthcare Journal. 2020;7(1):60-4.
10. Chan B, Edwards ST, Mitchell M, Nicolaidis C, Kansagara D, Korthuis PT, et al. Can an intensive ambulatory care intervention improve the experience of high-utilizing patients?: 6-month outcomes of the summit randomized controlled trial. Journal of General Internal Medicine. 2019;34(2 Supplement):S151.
11. Chang BP, Rostanski S, Willey J, Miller EC, Shapiro S, Mehendale R, et al. Safety and Feasibility of a Rapid Outpatient Management Strategy for Transient Ischemic Attack and Minor Stroke: The Rapid Access Vascular Evaluation-Neurology (RAVEN) Approach. Annals of Emergency Medicine. 2019;74(4):562-71.
12. Cornett L, Davidson S, McElvanna K. Ambulatory ultrasound scans reduce inpatient admissions during COVID-19: A need for the expansion of ambulatory services. British Journal of Surgery. 2021;108(SUPPL 7):vii134.
13. Elbehairy AF, McIsaac H, Hill E, Norman PA, Day AG, Neder JA, et al. Impact of a Specialized Ambulatory Clinic on Refractory Breathlessness in Subjects With Advanced COPD. Respiratory Care. 2020;65(4):444-54.

14. Fay LN, Wolf LM, Brandt KL, DeYoung GR, Anderson AM, Egwuatu NE, et al. Pharmacist-led antimicrobial stewardship program in an urgent care setting. American Journal of Health-System Pharmacy. 2019;76(3):175-81.
15. Greensitt B. Specialist physiotherapist leading in the frailty revolution in Ambulatory Emergency Care at the John Radcliffe Hospital, Oxford. Age and Ageing Conference: British Geriatrics Society Autumn Meeting, BGS. 2019;49(Supplement 1).
16. Hand BN, Boan AD, Bradley CC, Charles JM, Carpenter LA. Ambulatory Care Sensitive Admissions in Individuals With Autism Spectrum Disorder, Intellectual Disability, and Population Controls. Autism research : Official Journal of the International Society for Autism Research. 2019;12(2):295-302.
17. Hohman JA, Patel A, Parikh P, Rothberg MB. Comparing Encounter Characteristics Among Advanced Practice Clinicians and Physicians for Adult Same-Day Visits in Primary and Urgent Care. Journal of General Internal Medicine. 2022;37(3):689-91.
18. Lakhani PM, Ali S, Sames E, Agarwal P, Lisk R. Designing a Fast-Track Pathway for the Diagnosis of Giant Cell Arteritis in a Busy Ambulatory Emergency Care Unit Based on British Society of Rheumatology Guidelines. Rheumatology (United Kingdom). 2022;61(SUPPL 1):i30.
19. Magee MF, Baker KM, Fernandez SJ, Huang CC, Mete M, Montero AR, et al. Redesigning ambulatory care management for uncontrolled type 2 diabetes: a prospective cohort study of the impact of a Boot Camp model on outcomes. BMJ Open Diabetes Research & Care. 2019;7(1):e000731.
20. Raaber N, Botker MT, Riddervold IS, Christensen EF, Emmertsen NC, Grofte T, et al. Telemedicine-based physician consultation results in more patients treated and released by ambulance personnel. European Journal of Emergency Medicine. 2018;25(2):120-7.
21. Saleh A, Eltilib I, Sayed R, Peter M, Dobbins BM, Saha A. Surgical Ambulatory Emergency Care: Safe and Effective Management Route for Acute Surgical Patients. Journal of the American College of Surgeons. 2019;229(4 Supplement 2):e23-e4.
22. Standing H, Boag K, Hughes M, Amtul N. Is an ambulatory emergency general surgery management pathway of biliary disease more cost effective than a traditional approach? A matched case series study. British Journal of Surgery. 2021;108(SUPPL 9):ix50.

No relevant outcomes (n=13)

1. Abdulazeez Z, Tran U. Ambulatory Emergency Care Unit (Aec) in Patients' and Healthcare Professionals' Eye. BMJ Leader. 2020;4(Supplement 1):A43.
2. Atkin C, Knight T, Subbe C, Holland M, Cooksley T, Lasserson D. Response to winter pressures in acute services: analysis from the Winter Society for Acute Medicine Benchmarking Audit. Bmc Health Services Research. 2022;22(1).
3. Chidambranath R, Thomas P, Flatt J. Review of acute referrals to Surgical SDEC at a DGH. British Journal of Surgery. 2022;109.
4. Cooksley T, Knight T, Lindsay D, Gupta A, Ho JH, Higham C, et al. Immune checkpoint inhibitor-mediated hypophysitis: no place like home. Clinical Medicine. 2023;23(1):81-4.

5. Dave A, Ofili-Yebovi D, Cavill S. The case for change of a clinical pathway for ambulatory hyperemesis Gravidarum affected by the COVID-19 pandemic. BJOG: An International Journal of Obstetrics and Gynaecology. 2021;128(SUPPL 1):106.
6. Irvine N, Meer RV, Megiddo I. Early senior decision-making in acute medicine: a critical review of health policy and implications for practice. Acute Medicine. 2022;21(3):126-30.
7. Kroll DS, Wrenn K, Grimaldi JA, Campbell L, Irwin L, Pires M, et al. Impact of a Rapid-Access Ambulatory Psychiatry Encounter on Subsequent Emergency Department Utilization. Community Mental Health Journal. 2021;57(5):973-8.
8. Madge O, Bowen J. Undiagnosed malignancy presenting to same-day emergency care: a single unit experience. Clinical Medicine. 2022;22:S62-S3.
9. O'Neill A, Harrhy L. Reducing attendance at trauma clinics by providing first-point-of-contact treatment. Emergency Nurse. 2019;27(4):25-9.
10. Sene PM, Yahiaoui D, Koolian M, Tagalakis V. The Diagnostic Performance of a Non-Emergency Department-Based Deep Vein Thrombosis Ambulatory Care Pathway. Thrombosis Research. 2019;182(Supplement 1):8.
11. Sultan A, Hussain MI, Sellahewa C, Bird J, Glover G. Surgical Same Day Emergency Care at a District General Hospital - Our Experience. British Journal of Surgery. 2022;109.
12. Swamad M, Badawi M, Vitkauskas R, Cutcu D, Klimovskij M. Improving Acute Surgical Services and Establishing Same Day Emergency Care Clinic in a District General Hospital. British Journal of Surgery. 2022;109.
13. Wang BX, Reddy R, Gothandaraman B. An Ambulatory Syncope Service is a Safe Pathway to Reduce Hospital Admissions. Circulation Conference: American Heart Association's. 2022;146(Supplement 1).

Wrong publication type (editorials, letters, protocols, reviews) (n=11)

1. Atkin C, Riley B, Sapey E. How do we identify acute medical admissions that are suitable for same day emergency care? Clinical Medicine. 2022;22(2):131-9.
2. Cooksley T, Klotz A, Marshall E, Weaver J, Font C, Lasserson D. The need for ambulatory emergency oncology: exemplified by the management of immune checkpoint inhibitor toxicity. Supportive Care in Cancer. 2023;31(12):653.
3. Cooksley T, Marshall W, Ahn S, Lasserson DS, Marshall E, Rice TW, et al. Ambulatory emergency oncology: A key tenet of future emergency oncology care. International Journal of Clinical Practice. 2020;74(1):e13436.
4. Cottrell E, Mallen CD, Lasserson DS. Ambulatory emergency care: how should acute generalists manage risk in undifferentiated illness? British Journal of General Practice. 2018;68(666):12-3.
5. Dean S, Barratt J. What is the existing evidence base for adult medical same day emergency care in UK NHS hospitals? A scoping review protocol. Bmj Open. 2023;13(10).
6. Houghton M. Same-day emergency care. Clinical Medicine. 2023;23(1):99-.
7. Iacobucci G. Plans for same day emergency care are being "grossly derailed" by winter pressures. Bmj-British Medical Journal. 2020;368.

8. Mangiapane S, Czihal T, Stillfried DV. The Utilization of Ambulatory Emergency Care and Unplanned Hospitalizations in Germany, 2010-2019. Deutsches Arzteblatt International. 2022;119(24):425-6.
9. O'Dowd A. All neighbourhoods need "single, urgent care teams" offering same day access, says review. BMJ. 2022;377:o1342.
10. Stabile C, Temple LK, Ancker JS, Basch E, Carter J, Miranda M, et al. Ambulatory cancer care electronic symptom self-reporting (ACCESS) for surgical patients: a randomised controlled trial protocol. BMJ open. 2019;9(9):e030863.
11. Thompson D, Connolly V. Ambulatory emergency care. British Journal of Hospital Medicine. 2018;79(1):6-7.

Wrong patient group (n=3)

1. Al Sayari RA, Hussain Z, Engledow A. The affect of an emergency ambulatory care centre (ESAC) on the pre-operative inpatient stay prior to appendicectomy. British Journal of Surgery. 2019;106(Supplement 5):93.
2. Alvarado G, Hegg L, Rhodes K. Improving Psychiatric Access for Students in Crisis: An Alternative to the Emergency Department. Psychiatric Services. 2020;71(8):864-7.
3. Goh OQM, Xin X, Lim WT, Tan MWJ, Kan JYL, Osman HB, et al. Economic Evaluation of Novel Models of Care for Patients With Acute Medical Problems. JAMA Network Open. 2023;6(9):e2334936-e.

Duplicate study or study data (n=5)

1. Atkin C, Knight T, Cooksley T, Holland M, Subbe C, Kennedy A, et al. Society for Acute Medicine Benchmarking Audit 2021 (SAMBA21): assessing national performance of acute medicine services. Acute medicine. 2022;21(1):19-26.
2. Baker J. Effects of ambulatory emergency care on organisational and patient outcomes. Nursing Management (Harrow). 2018;25(4):36-41.
3. Sene PM, Yahiaoui D, Koolian M, Tagalakis V. The Diagnostic Performance of a Non-Emergency Department-Based Deep Vein Thrombosis Ambulatory Care Pathway. Blood. 2019;134(Supplement 1):3405.
4. Eltilib I, Sayed R, Saleh A, Rees B, Dobbins B, Saha A. Surgical ambulatory emergency care: A safe and effective management route for acute surgical patients. British Journal of Surgery. 2019;106(Supplement 5):108.
5. Greensitt B, Bowen J, Singh S, Vaziri L, Bambarvajane A, Price J, et al. The role of the specialist physiotherapist in ambulatory emergency care; leading on developing the frailty pathway in the ambulatory assessment unit at the John Radcliffe Hospital, Oxford. Future Healthcare Journal. 2019;6(Suppl 2):41.

Appendix 4

Table 3 Review outcomes for SDEC and urgent and emergency care delivery.

First author, year	Discharge (as reported)	Mortality (30-day or other timepoint as reported)	SDEC referral / appropriateness	Inpatient Admission (as reported)
Ali & Karmani, 2018[27]	Same day: 'More than 92% ….'; 'zero day discharges increased to 15%'	-	50% of the 8% of the patients admitted to the AAU 'referred inappropriately'	'Only 8% of the patients were admitted to the Acute Admission Unit'
Atkin et al, 2022[19]	Same day (2021): ED, AMU, SDEC (and 'other') overall rate: 31.5%	-	-	-
Atkin et al, 2023[18]	Same day (2022): SDEC 82.1% (1135/1382) vs ED, AMU, SDEC overall 28.9%	14-day: ED 4.5% vs AMU 1.8% vs SDEC 0% ($p<0.005$)	-	Overall: 1.1%; SDEC 0.1% vs AMU 0.7% ($p = 0.015$) or ED 1.7% ($p<0.001$)
Baker at al, 2018[13]	SDEC 2014: 200/200 (100%) vs ED 2013: (n=124/191, 65%)	-	-	SDEC 2014: 0/200 (0%) vs *ED 2013: 67/191 (35%) ($p<0.001$)
Baker et al, 2019[20]	Overall same day: 95% (SDEC or ED/SDEC)	-	-	-
Balaratnam et al, 2022[24]	-	-	-	-
Byrne & McCall, 2018[15]	-	30-day: 4/946 (0.4% of patients seen in the ACU). 'None of these patients died from the original cause for referral to the ACU'	-	13.5% decrease in cardiology admissions. From ED: fell from 1,651/86,520 attendances (baseline) to 1,428/93,221 attendances (1 year post ACU initiation). Among unscheduled care representations, 25/60 patients were admitted to hospital from the ED (41.6% of reattenders, 2.6% of all patients seen). Decreased admissions in conditions targeted by the ACU: 24% for AF and other atrial arrhythmias; 29% syncope; 45% pericarditis; no reduction for HF or palpitations
Corvan et al, 2022[16]	'Following appropriate same day investigations, 66% of patients were suitable for	-	First 3 months of ACU, 137 patients were referred from ED … 90% of referrals were accepted (met criteria)	Post-intervention: '15% reduction in the number of patients admitted to Cardiology and a 24% reduction in AMU referrals to Cardiology'

First author, year	Discharge (as reported)	Mortality (30-day or other timepoint as reported)	SDEC referral / appropriateness	Inpatient Admission (as reported)
	discharge with a diagnosis and management plan’			
Edison et al, 2021[22]	‘Primary rate’ 38.3% (n=67 including 6 DNAs); ‘overall discharge rate’ (including a follow-up SDEC appointment) 84% (n=147)	-	‘Nine referrals (5.1%) were inappropriate and did not meet AEC criteria, however, of these only one required admission for observation’	28 (16.0%) of which 18 (10.3%) were ‘clinically appropriate’; for 10 (5.7%) patients, admission could have been avoided
Elias et al, 2021[29]	-	30-day: 31 (6%). At 1 year: 138 (28%)	-	39% (of which immediate 72%; within 30 days 28%). Unplanned admissions within 30 days: 58 (11%)
Hsu et al, 2022[30]	A quarter discharged home after specialist review… 61% no need to re-attend SDEC**	-	-	None required admission from clinic (n=255)**
Jarral et al, 2020[25]	Same day 31/33 (93.93%)	-	In retrospect, 11 admitted patients would have been suitable for SDEC as part of direct referrals pilot	20 patients, specifically with suspected PE, were admitted from the ED
Keane et al, 2022[14]	6.3% reduction inpatients discharged from the whole study site with <24 hours LOS, based on the last month versus first 6 months of operation (average reduction of 118 to 114 patients). HSPT >25% reduction	-	8.39% of all ED presentations referred to SDEC (range 10–40 patients/day). ‘Did not wait’: Average 1.30% (SDEC) vs 6.18% (ED): HSPT ⩽5.23%	SDEC admitted 960 patients to an inpatient multiday bed; average admission conversion rate (first six months): 18%, reduced in Aug to an average of 7.65% (10.35% reduction). Daily admission rates ranged from 0% to 42%. HSPT ⩽10%
Keaney et al, 2019[31]	-	-	‘10% of all referrals for repeat or follow-up blood tests’; Week 1 rejected new referrals (by SDEC consultant): (31%); 4 weeks post-opening: 18%	-
Pincombe et al, 2023[17]	-	-	-	ED-referred: ‘There were no changes predicted in either the level or trend of hospital admissions following the first MACS appointment for the ED-referred group'; results consistent for 12- and 18-months pre-MACS analyses

First author, year	Discharge (as reported)	Mortality (30-day or other timepoint as reported)	SDEC referral / appropriateness	Inpatient Admission (as reported)
Ray et al, 2020a[23], 2020b[12]	'Discharge rate' increased from 60% to 72% . Number of completed discharge summaries increased to 94% (n=47) from 82% (n=41)	-	n=100 (50 in audit 1; 50 in audit 2): Managed in and appropriate for SDEC (increased from 38% to 54%); not managed in and not appropriate (decreased from 38% to 18%); not managed in SDEC but appropriate for SDEC (unchanged 6 %); managed in SDEC but not appropriate for SDEC (increased from 18% to 22%)	Inpatient admission following AEC review reduced from 40% (n=20) to 28% (n=14)
Reddy et al, 2022[26]	-	-	~10% (13/136) were triaged towards ambulatory care	~10% (13/136) post-pathway patients avoided hospital admission
Reschen et al, 2020[21]	74.8% single visit; 14.0% visits spanned 2 consecutive days	30-day over the 38-month period: SDEC 1.6%; non-ambulatory pathway: 8%. Over 3 years: SDEC 14.6%; EAU: 28.9%	The proportion of acute medical referrals to SDEC increased to 42% (mean 700 referrals seen per month, last 6 months of study); numbers seen in the non-ambulatory pathway fell	38-month period: Mean conversion to admission: SDEC (13.8%) vs EAU (54.9%). SDEC 'admission rate' fell from 16.6% in the first full calendar year to 12% in the last calendar year
Visanji et al, 2020[11]	-	-	Previously '50% of the GP expected patients were seen by the medical take via the ED with 9% being reviewed in SDEC; after the AMU opened 47% were seen in SDEC with only 7% being reviewed in ED'	-
Weihser et al, 2018[28]	3-months: overall 8.2% increase in same-day discharges from acute medical unit	30-day baseline: 4.5% of attendances at the old service. Post-service introduction: 0.7%	-	Baseline: Not reported. 3-month pilot: 11.9% (month 1), 14.7% (month 2), 18.2% (month 3). 12-month study average: 6.1%

*Visual estimate from bar chart (Figure 3) and the 'approximately two-thirds' cited in the text; **There is a lack of clarity in the data as reported

AAU: Acute Admission Unit; ACU: Ambulatory Cardiology Unit; AEC: Ambulatory Emergency Care: AF: Atrial fibrillation; AMU: Acute Medical Unit; CI: Confidence Interval; DNA: Did Not Attend; EAU: Emergency Assessment Unit; ED: Emergency Department; GP: General Practitioner; HF: Heart failure; HSPT: Health Service Performance Target; LOS: Length of stay; MACS: Medical Ambulatory Care Service; N-SDEC: Neurology Same Day Emergency Care; PE: Pulmonary embolism; SDEC: Same Day Emergency Care

Table 4 Further reported outcomes for SDEC and urgent and emergency care delivery.*

First author, year	ED or SDEC reattendance (30-day or other timepoint as reported)	Onward referral	Inpatient bed occupancy / Saved bed days	Cost
Ali & Karmani, 2018[27]	-	-	-	-
Atkin et al, 2022[19]	Unscheduled within 7 days (overall): '2.5% returned via ED attendance, 1.8% via another route including AMU'	-	-	-
Atkin et al, 2023[18]	-	-	-	-
Baker at al, 2018[13]	-	-	-	-
Baker et al, 2019[20]	Overall, 0% (of SDEC or ED and SDEC)	Overall: 5% (of SDEC or ED and SDEC)	-	-
Balaratnam et al, 2022[24]		'Only 5% of referrals to N-SDEC required onward General Neurology outpatient services'	-	-
Byrne & McCall, 2018[15]	Unscheduled (30-day) 60/946 patients (6.3% of all patients seen in the ACU; 8=ACU, 52=ED). 6701 more ED attendances, Sep 2015 to Aug 2016	-	-	Increased ED admissions estimated in 1 year, based on increased ED attendances all leading to 1-night admissions: £111,500
Corvan et al, 2022[16]	ACU post intervention: <2% (and no adverse patient outcomes at 30 days)	-	400 bed days saved	Projected £860,000 savings in the first year of service (based on saved bed days)
Edison et al, 2021[22]	Overall SDEC follow-up appointments not reported	-	-	147 patients avoided admission over 3-month period. Cost of a non-elective admission for abdominal pain without intervention is £610; cost of a non-elective admission for abdominal pain with intervention is £2366
Elias et al, 2021[29]	-	-	-	-
Hsu et al, 2022[30]	Overall SDEC reattendance not reported	-	-	-

First author, year	ED or SDEC reattendance (30-day or other timepoint as reported)	Onward referral	Inpatient bed occupancy / Saved bed days	Cost
Jarral et al, 2020[25]	-	-	-	-
Keane et al, 2022[14]	<48 h: Average 1.51% (SDEC) vs 3.59% (ED). HSPT ⩽2.73%	-	-	-
Keaney et al, 2019[31]	-	Week 1 to other clinics and specialties: 30%. At 4-weeks post-opening: 19%	-	-
Pincombe et al, 2023[17]	-	-	-	MACS was found to be cost-effective for GP (total cost of ED presentations/100 patients in 30 days estimated to fall by −$1018 [95% CI:−$2,272 to $236]) and ward-referred (trend predicted to decrease by −$5221 [95% CI: −$8150 to −$2292]) groups, but expected impact for ED-referred sensitive to assumptions
Ray et al, 2020a[23], 2020b[12]	-	-	-	-
Reddy et al, 2022[26]	-	-	-	-
Reschen et al, 2020[21]	30-day, 3rd year: SDEC 6.9%; non-ambulatory pathway: 18.0%	-	Mean overnight patients from: SDEC 13.9; non-ambulatory pathway: 165.4. The number from SDEC remained constant over 3 years, despite more patients seen on SDEC	-
Visanji et al, 2020[11]	-	-	-	-
Weihser et al, 2018[28]	-	-	8.9% decrease in 1-, 2- and 3-day LOS patient admissions – equating to 59 saved bed days during the pilot period	-

*Excluding ED wait times/LOS reported previously (Carroll C, Kundakci B, Muhinyi A, et al., 2024).

ACU: Ambulatory Cardiology Unit; AEC: Ambulatory Emergency Care: AF: Atrial fibrillation; AMU: Acute Medical Unit; CI: Confidence Interval; DNA: Did Not Attend; EAU: Emergency Assessment Unit; ED: Emergency Department; GP: General Practitioner; HSPT: Health Service Performance Target; LOS: Length of stay; MACS: Medical Ambulatory Care Service; N-SDEC: Neurology Same Day Emergency Care; PE: Pulmonary embolism; SDEC: Same Day Emergency Care
